# Fang Filling

**Published:** 1863-01

**Authors:** 


### FANG FILLING.

("Monsieur Tonson come again.")

A CALM, intelligent patient, in a lunatic asylum, was asked by a visitor how it happened that he was an inmate. "A triumph of our republican institutions, sir," was the patient's reply. "I thought all the world had gone mad;—all the world entertained a like view of me. So I was *outvoted;* and here I am. All right, sir, and according to the Constitution!"

But what about fang filling? Only this: An esteemed brother enquires, "Do you still prepare nerve cavities for filling, and then let them go?" To which our answer must be partly *yes*, and considerably *no.* We fill more fangs than we did a year or two ago, and will try to tell why:—

We can prepare a canal for filling in less time than we can get it ready to leave unfilled. Nature must have time to close up the orifice at the end of the root. If the surroundings are healthy, we plug up the oriffce at once with gold as soon as the canal is thoroughly cleaned out.

We like to fill canals. It arrests the attention, requires some thought, and some nice manipulations. We never did enjoy handwork that requires but little *headwork.*

Some patients prefer to have the fangs filled; and we like to gratify them—like to gratify our friends in everything that is not unreasonable; and fang filling is not. This last phrase amounts to a public abandonment of a formerly expressed notion, that the conducting property of the gold in the canal predisposes to *periostitis.*

But the principal reason is the one suggested in our preface. We have been *outvoted.* A majority of the best educated members of the profession fill fangs for reasons they consider good. A man is not safe in arraying himself against the opinions of the whole profession. Before he does so he should "look well to his going." We felt our way cautiously before making a public expression of opinion on the subject, and before adopting *non-fang filling* as our rule of practice. But fearing that the profession might be *wiser than we*, we resumed fang filling to a considerable extent, however, practising the other treatment in a number of cases sufficient for extended and thorough observation. And so far we have not found anything to shake our confidence in *non-fang filling*.

When we do fill fangs we aim to fill thorougly. If it will not do to leave the canal unfilled, it can do but little better to fill it so loosely that the gold can be withdrawn to see how it is doing, or for further treatment of the tooth. Nor is the filling with plaster of Paris or gun cotton any better. If the danger is from "serum in the pulp-cavities," gun cotton, plaster, and loose fragments of gold will not keep it out. W.

